# Efficacy of artesunate-amodiaquine and artemether-lumefantrine fixed-dose combinations for the treatment of uncomplicated *Plasmodium falciparum* malaria among children aged six to 59 months in Nimba County, Liberia: an open-label randomized non-inferiority trial

**DOI:** 10.1186/1475-2875-12-251

**Published:** 2013-07-17

**Authors:** Birgit Schramm, Parastou Valeh, Elisabeth Baudin, Charles S Mazinda, Richard Smith, Loretxu Pinoges, Mehul Dhorda, Yap Boum, Timothy Sundaygar, Yah M Zolia, Joel J Jones, Eric Comte, Pascal Houzé, Vincent Jullien, Gwenaelle Carn, Jean-René Kiechel, Elizabeth A Ashley, Philippe J Guérin

**Affiliations:** 1 Epicentre, 75011 Paris, France; 2 Epicentre Mbarara Research Base, Mbarara, Uganda; 3 National Malaria Control Programme, Ministry of Health and Social Welfare, Monrovia, Liberia; 4 Médecins Sans Frontières, 1211 Geneva, Switzerland; 5 AP-HP, Hôpital St-Louis, Laboratoire de Biochimie, 75010 Paris, France; 6 INSERM U663, Université Paris Descartes, 75006 Paris, France; 7 Drugs for Neglected Diseases initiative, 1202 Geneva, Switzerland; 8 Centre for Tropical Medicine, Nuffield Department of Clinical Medicine, University of Oxford, CCVTM, Oxford, UK

**Keywords:** Malaria, Artemisinin, Efficacy, Randomized trial, Liberia

## Abstract

**Background:**

Prospective efficacy monitoring of anti-malarial treatments is imperative for timely detection of resistance development. The *in vivo* efficacy of artesunate-amodiaquine (ASAQ) fixed-dose combination (FDC) was compared to that of artemether-lumefantrine (AL) among children aged six to 59 months in Nimba County, Liberia, where *Plasmodium falciparum* malaria is endemic and efficacy data are scarce.

**Methods:**

An open-label, randomized controlled non-inferiority trial compared the genotyping adjusted day 42 cure rates of ASAQ FDC (ASAQ Winthrop®) to AL (Coartem®) in 300 children aged six to 59 months with uncomplicated falciparum malaria. Inclusion was between December 2008 and May 2009. Randomization (1:1) was to a three-day observed oral regimen (ASAQ: once a day; AL: twice a day, given with fatty food). Day 7 desethylamodiaquine and lumefantrine blood-concentrations were also measured.

**Results:**

The day 42 genotyping-adjusted cure rate estimates were 97.3% [95% CI: 91.6-99.1] for ASAQ and 94.2% [88.1-97.2] for AL (Kaplan-Meier survival estimates). The difference in day 42 cure rates was −3.1% [upper limit 95% CI: 1.2%]. These results were confirmed by observed proportion of patients cured at day 42 on the per-protocol population. Parasite clearance was 100% (ASAQ) and 99.3% (AL) on day 3. The probability to remain free of re-infection was 0.55 [95% CI: 0.46-0.63] (ASAQ) and 0.66 [0.57-0.73] (AL) (p = 0.017).

**Conclusions:**

Both ASAQ and AL were highly efficacious and ASAQ was non-inferior to AL. The proportion of patients with re-infection was high in both arms in this highly endemic setting. In 2010, ASAQ FDC was adopted as the first-line national treatment in Liberia. Continuous efficacy monitoring is recommended.

**Trial registration:**

The protocols were registered with Current Controlled Trials, under the identifier numbers ISRCTN51688713, ISRCTN40020296.

## Background

Despite notable decreases in malaria incidence rates in various endemic settings, *Plasmodium falciparum* malaria remains a serious public health problem especially for the African continent [1]. Potent artemisinin-containing combination therapy (ACT) is the recommended first-line treatment for uncomplicated falciparum malaria in most endemic countries [2]. This includes artesunate-amodiaquine (AS + AQ), artemether-lumefantrine (AL), artesunate-mefloquine (AS-MQ), artesunate-sulfadoxine-pyrimethamine (AS-SP) and dihydroartemisinin-piperaquine (DHAPQ), which are considered efficacious and safe [2-5]. Fixed-dose combinations (FDC) should be used whenever possible to support adherence. The first FDC of AS + AQ (ASAQ Winthrop®) was developed by the Drugs for Neglected Diseases initiative (DNDi), and is currently registered in 32 African countries. ASAQ FDC was shown to be efficacious, safe and well tolerated in previous studies [6-9].

Prospective periodic monitoring of the efficacy of anti-malarial treatments by *in vivo* studies is part of post-marketing surveillance [10]. Monitoring of ACT becomes particular important in the light of recent emergence of artemisinin resistance in South-East Asia [11-13]. In Liberia, the national treatment protocol for uncomplicated falciparum malaria was AS + AQ (separate tablets) since 2003, though only limited information was available on the efficacy of ACT in this highly malaria endemic country [14]. The present study assessed the *in vivo* efficacy of ASAQ FDC compared to that of artemether-lumefantrine in a non-inferiority trial among 300 children aged six to 59 months in Nimba County, a falciparum malaria endemic region in Northern Liberia. At the same site, both treatments were also studied in children over five years of age and adults in a parallel randomized trial with 28 days follow-up and the primary objective to obtain data on the safety and tolerability in this under-studied age group. The methods and findings of this second trial are reported in detail elsewhere [15] but a summary of efficacy results in the older age group are included here.

## Methods

### Study site, objectives and design

An open-label, randomized non-inferiority trial was carried out between December 2008 (first inclusion) and July 2009 (last scheduled follow up visit) in the Comprehensive Healthcare Center (CHC) of Saclepea, Nimba County. The facility was supported by Médecins Sans Frontières and the Ministry of Health and served the local population and displaced persons from Ivory Coast in a nearby refugee camp. The primary objective was to evaluate the efficacy of amodiaquine-artesunate versus artemether-lumefantrine among children between six and 59 months old suffering from uncomplicated malaria defined as the genotype adjusted cure rates at day 42 [10]. Secondary objectives were: (a) to evaluate the genotype- unadjusted cure rates at day 42, genotype adjusted/unadjusted cure rates at day 28, and re-infection rates at day 42; (b) to assess blood drug concentration differences possibly influencing efficacy by measuring drug concentrations of amodiaquine and lumefantrine at day 0 and day 7; (c) to assess the safety of amodiaquine-artesunate and artemether-lumefantrine treatment among children between 6 and 59 months by documenting adverse events that occurred during the study before day 28 and by documenting serious adverse events; (d) to formulate recommendations for adapted case management of malaria in Nimba County. The findings on tolerability and safety (secondary objective (c)), are described in detail elsewhere [15].

Inclusion criteria were: age six to 59 months; fever (axillary temperature ≥ 37.5°C) or fever in previous 48 hours; blood smear-confirmed asexual stages of falciparum malaria (*P. falciparum* mono-infection) and parasite density between 2,000–200,000/μl blood; high probability of attending follow-up; signed informed consent (patient or responsible caregiver). Exclusion criteria were: severe/complicated malaria [16]; severe anaemia (<5 g/dl haemoglobin (Hb)); full course of AS + AQ or AL in the past 10 days; known hypersensitivity to the study drugs; concomitant febrile illness other than malaria that may confound outcome; severe malnutrition (weight-for-height < 70% of median and/or symmetrical edema involving at least the feet). Previous intake of anti-malarials other than AS + AQ or AL did not lead to exclusion from study participation, Study visits were on Days 0, 1, 2, 3, 7, 14, 21, 28, 35 and 42. Patients were not routinely hospitalized for study participation unless required for treatment of any clinical presentations during study follow up. All routine measures that were performed during study visits are described below.

### Randomization, drug allocation and blinding

Inclusion was between December 2008 and May 2009. Patients HRP-2 rapid diagnostic test-positive (Paracheck®) were screened for inclusion by clinical examination, malaria blood smear, Hb measure (capillary blood, HemoCue®). Eligible patients were randomized at 1:1 ratio to ASAQ or AL. Random allocation (computer-generated, block size of 6, study site unaware of block size) was provided in sealed, opaque individual envelopes which were opened in consecutive order by study nurses at drug allocation. Allocation was not disclosed to the medical staff performing the clinical exams, but different intake schedules of ASAQ and AL may have compromised full masking. The laboratory team was un-blinded to treatment allocation since blood spots for lumefantrine concentration assessment (AL arm) were collected on filter-paper pre-treated with tartaric acid.

### Treatment

Treatment by study arm was on days 0, 1 and 2. ASAQ (ASAQ Winthrop®, Sanofi-Aventis) was given once a day without co-administration of food, though breast feeding for infants in the ASAQ arm around the time of drug intake was not discouraged. AL (Coartem®, Novartis) was given as two doses per day, 6–12 hours between doses, and administered with a high-fat cookie. For small children the cookie was given as a paste mixed with water or breast-feeding was encouraged. Both treatments were administered with a glass of water. For infants who could not swallow tablets, tablets were dissolved (ASAQ) or crushed and mixed (AL) with a small volume of water. Both treatments were three-day oral regimens dosed by weight according to the manufacturer’s instructions: ASAQ Winthrop® 5 to <9 kg: one tablet/day of artesunate (AS) 25 mg/amodiaquine (AQ) 67.5 mg; 9 to <18 kg: one tablet/day of AS 50 mg/AQ 135 mg; 18 to <36 kg: 1 tablet/day of AS 100 mg/AQ 270 mg; ≥36 kg: 2 tablets/day of AS 100 mg/AQ 270 mg. Coartem® tablet strength was 20 mg artemether/120 mg lumefantrine: 5 to <15 kg: 1 tablet/dose; 15 to <25 kg: 2 tablets/dose; 25 to <35 kg: 3 tablets/dose; ≥ 35 kg 4 tablets/dose. The children’s weight was rounded to the nearest kg for dosing of the study drug. All doses were administered in the study site and observed for 30 minutes. If vomited/spat-out within 30 minutes, a full-dose was re-administered. If again vomited/spat-out within 30 minutes, the patient was withdrawn and rescue treatment given (parenteral quinine or intramuscular artemether). In the event of treatment failure and in the absence of signs of severe malaria, patients in the AL arm were treated with ASAQ Winthrop, and patients in the ASAQ arm were treated with AL (administered with a fatty cookie) and terminated from further study follow-up. The first dose of rescue treatment was given in the study site, observed for 30 minutes and re-administered in case of vomiting.

### Blood smear follow up and parasite genotyping

Giemsa-stained malaria thin and thick blood smears were prepared from capillary blood on days 0, 2, 3, 7, 14, 21, 28, 35 and 42. Trophozoites were counted against 500 white blood cells (WBCs) in the thick smear, assuming an average of 8000 WBCs / μl blood. For high parasitaemia, parasites were counted against 1000 red blood cells in the thin smear. Plasmodium species and parasite blood stages were differentiated on the thick smear in at least 30 high power fields, and confirmed on the thin smear. All blood smears were read by a second reader blinded to the first reader’s result. Readings of two readers in agreement were reported as final result. For discrepant slides the final reading was by the lab supervisor or a designated senior reader. External quality control (EQC) was performed by the Shoklo Malaria Research Unit (SMRU) in Mae Sot, Thailand, on 10% of all day 0 enrolment slides, 10% of all day 2 slides, 10% of all negative follow-up slides and all failure slides.

For each patient at enrolment, and on any follow up day with blood-smear-confirmed recurrent parasitaemia, two blood spots were prepared from capillary or venous blood (in EDTA) onto filter paper (Whatman FTA®cards) for parasite genotyping to distinguish between falciparum malaria recrudescence and re-infection, using three genetic markers (*glurp, msp1, msp2*) as described elsewhere [17]. In the second trial on the tolerability of ASAQ and AL among 1000 patients aged >5 years with uncomplicated falciparum malaria (1:1 randomized to ASAQ or AL, details reported elsewhere) the malaria blood smear follow up was limited to days 0, 2 and 28, day 3 if positive on day 2 [15], and any visit if fever.

### Clinical monitoring and laboratory follow up

At each visit a standardized symptoms questionnaire and physical examination were conducted by trained study physician assistants. Vital signs (pulse rate, respiratory rate, and blood pressure) were recorded on day 0 and 28 for all patients. Fever was assessed at every visit by measuring the axillary temperature with a digital thermometer. Temperatures > 37.5°C were recorded as fever. Haemoglobin (Hb) was measured on each visit (finger-prick capillary blood, HemoCue®), and liver function tests were conducted by assessment of blood levels of aspartate aminotransferase (AST) and alanine aminotransferase (ALT) (Reflotron plus®, Roche Diagnostics) on days 0 and 28. A full blood count (FBC) was done on days 0, 7 and 28 (Act5diff, Beckman Coulter®), or if the HemoCue® result indicated anaemia (defined as <10 g/dl Hb for children < 2 years, <11 g/dl Hb for children > 2 years) [18]. Clinical- or laboratory signs and symptoms which occurred or worsened at any time after the first drug intake up to day 28 were recorded as adverse events (AEs) to document tolerability and safety (for details on adverse event recording and results [15].

### Sample size

The sample size calculation was based on an assumed efficacy of 96% for both ACTs, a non-inferiority margin of 6%, a power of 80%, and a one-sided 5% significance level. To this end 132 children per study arm were required (nQuery Advisor). With a 10% lost to follow up rate, the total number of children was rounded to 300 (150 per study arm).

### Efficacy outcome analysis

Efficacy analyses were conducted on both mITT (modified-intention-to-treat) and PP (per protocol) populations. The mITT population was defined as all randomized patients with parasitological confirmation of falciparum malaria at day 0 who took at least one dose of study drug. The PP population was defined as all patients who were included into the mITT population and who did not have a major protocol deviation and who were not pre-maturely discontinued from the study for reasons other than an adverse event (AE) or recurrence of parasitaemia. Major deviations were defined as: i) at inclusion: weight < 5kg, no microscopic confirmation of asexual stages of Pf malaria or mixed infection, asexual parasites density < 2,000 or > 200,000 /μl of blood, no fever (axillary temp > 37.5°C) or no history of fever in the last 48 hours, no informed consent, presence of general danger signs, signs of severe/complicated malaria, severe anaemia (Hb < 5g/dL), severe malnutrition (WH < 70%), having received a full course of the treatment under study in the previous 10 days; ii) during study conduct: any dose of study drug not taken or vomited and not replaced / or delayed by ≥ 1 calendar date, two consecutive study visits not performed in the allowed time window [−1; +3 days], or intake of concomitant medication (except for anti-pyretics) during follow-up if medication is of known anti-malarial activity. Results were reported by treatment group as allocated. Treatment outcomes were classified using standard definitions: adequate clinical and parasitological response (ACPR), early treatment failure (ETF), late parasitological failure (LPF), and late clinical failure (LCF) [10]. The main efficacy endpoints were day 42-genotyping-adjusted cure rates. Secondary efficacy endpoints were genotyping-adjusted day 28, and genotyping-unadjusted day 28 and day 42 efficacy outcome. Day 42 and day 28 cure rates were provided through estimates of survival by Kaplan-Meier (KM) analysis on the mITT population (mITT/KM), and by calculation of the proportion of patients cured by day 28 or day 42, respectively, with the PP population (PP/%). Patients with recurrent parasitaemia and missing or inconclusive genotyping results were excluded from genotyping-adjusted efficacy analyses and coded as failure for genotyping-unadjusted analyses (mITT/KM and PP/%) [10]. *P. falciparum* re-infections were censored at visit of recurrence for genotyping-adjusted mITT/ KM analysis, excluded from genotyping-adjusted PP/% analysis and coded as failure for all genotyping-un-adjusted analyses. Non-Pf malaria species infections were censored at visit of recurrence for mITT/KM analyses, and excluded from PP/% analyses. Pre-mature study discontinuations or patients with two consecutive scheduled visits not performed in the allowed time window [−1; +3 days] were censored at the last visit performed according to schedule for mITT/KM analysis. Pre-mature study discontinuation due AE were excluded from the PP/% analysis [10] (other premature discontinuations or patients with visits not performed in the allowed time window were already excluded from the PP population).

All proportions/rates were presented with a 95% confidence interval (CI) by treatment group. For assessment of non-inferiority of ASAQ versus AL, a 90% CI was constructed around the day 42 genotyping-adjusted cure rate difference (AL- ASAQ), and the upper limit of the CI (= a one-sided 95% CI) was compared to the pre-set non-inferiority margin. In addition, the Kaplan-Meier estimated probability of remaining free of *P. falciparum* re-infection (re-infection coded as failure, treatment failures or events without treatment outcome censored, patients with missing or undetermined genotyping excluded) was assessed per treatment arm and survival curves compared by log rank test. The median time to re-infection and interquartile range (IQR) per arm were assessed and the median compared using the Wilcoxon rank-sum test. The proportion of patients who achieved parasite clearance (asexual *P. falciparum*) on days 0, 2 and 3, and the proportion of patients with gametocytes on days 0, 2, 7, 14 and 28 were assessed. Analyses were performed with STATA 10.1. (Stata Corp, Texas), following a pre-set statistical analysis plan.

### Anti-malarial blood concentrations

Day 0 and day 7 blood concentrations of amodiaquine (AQ) and desethylamodiaquine (DEAQ) (ASAQ arm), or lumefantrine (LF) (AL arm), were assessed from venous blood (EDTA) preserved as dried spots on filter paper (Whatman® 31 ET). For LF samples the filter paper was pre-treated with tartaric acid (0.75 μM) and stored refrigerated [19]. Blood spots were sent once per month to the pharmacology laboratory in Paris and stored at −80°C until analysis. AQ, DEAQ and LF blood concentrations were measured by high performance liquid chromatography with ultraviolet detection (LF) or by tandem-mass spectrometry (AQ/DEAQ) with limits of quantification (LOQ) of <2.5 ng/ml (AQ), <5.0 ng/ml (DEAQ), and <200 ng/ml (LF), respectively. Day 7 samples collected before day 6 or after day 8 were excluded from statistical analysis on blood concentrations. Summary statistics were provided (mITT population), and comparison of day 7 blood concentrations between patients with day 42 efficacy outcome ACPR versus recrudescence, or ACPR versus re-infections, or ACPR versus combined *P. falciparum* recurrences (re-infection, recrudescence and recurrences with missing or undetermined genotyping results) respectively, performed (mITT, comparison by Wilcoxon rank-sum test). Furthermore, day 0 blood concentrations of other anti-malarials: pyrimethamine, sulfadoxine, chloroquine, quinine were retrospectively assessed from stored serum samples collected at baseline from each patient. Concentrations measures were by liquid chromatography coupled with tandem mass spectrometry, after protein precipitation by acetonitrile containing hydroxychloroquine as internal standard [20].

### Ethics

The procedures followed were in accordance with the ethical standards of the Helsinki Declaration. All participants or responsible caretakers signed informed consent. The studies were approved by the Liberian Institute for Biomedical Research (LIBR) Ethics committee, the Ministry of Health and Social Welfare, Monrovia, Liberia, and the Comité de Protection des Personnes (CPP) Ile de France XI (Saint Germain en Laye), France. The studies were registered at Controlled Trials (ISRCTN51688713, ISRCTN40020296).

## Results

### Inclusion, baseline characteristics and follow-up

**Figure 1:**
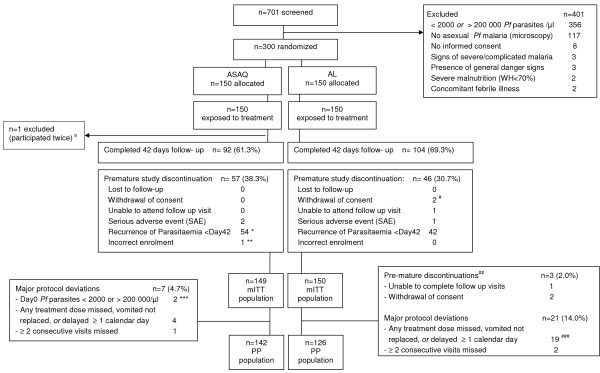
**Trial profile with mITT and PP populations.** RDT = rapid diagnostic test for malaria (Paracheck® ); *Pf* = *Plasmodium falciparum*; WH = Weight for Height; mITT = modified Intention to Treat; KM = Kaplan Meier; SAE = Serious Adverse Event; PP = Per Protocol. ^a^ Second participation excluded from analyses.* Note: one patient was discontinued with recurrent malaria (*Pf* re-infection, day 30). This patient is listed here among the n = 2 patients pre-maturely discontinued due to SAE (severe malaria). ** Patient was discontinued from participation on day 0 shortly upon enrolment because eligibility criteria were not met: i.e. asexual parasites density was outside the eligibility range (< 2000 or > 200 000 / μl blood). *** Two patients were enrolled with day 0 asexual parasites density outside the eligibility range (< 2000 or > 200 000 / μl blood): n = 1 patient was discontinued on day 0, once the eligibility error was discovered (discontinuation due to incorrect enrolment); n = 1 patient continued the follow-up to day 42. Both patients were excluded from the per-protocol population (protocol deviation). ^#^ Pre-mature discontinuations other than discontinuations due to adverse event or recurrence of parasitaemia which lead to exclusion from the PP population. ^##^ For n = 1 patient the care-taker withdrew consent on day 3; for n = 1 patient the care-taker withdrew consent on day 21. ^###^ n = 1 patient who also missed 2 evening doses is not listed among these n = 19, since the primary reason for exclusion of this patients from PP population was withdrawal of consent by care-taker (day 3) (patient listed among n = 2 consent withdrawn).

A total of 701 pre-screened patients were checked for eligibility, and 300 children were included and randomized to the two arms (Figure 1). Demographics and baseline parasitological- and clinical parameters were similar between the treatment arms (Table 1). The distribution of patients’ weight and age by weight-based drug dosage group and study arm are provided in Additional file 1. All randomized patients had been exposed to at least one dose of the study drug. All but one patient who participated twice (ASAQ arm, second participation excluded from all analyses) were included to the mITT population (n = 149 ASAQ, n = 150 AL) (Figure 1). Ninety-six-point-six percent (144/149) of patients (ASAQ, mITT) and 86.7% (130/150) of patients (AL, mITT), completed the study treatment according to schedule (no delay ≥ 1 calendar day, no dose missed). Delayed dose intake was recorded in the AL arm for 8.7% of patients (13/150, one or two doses delayed by one calendar day). The treatment of these patients was completed on day 3. Few patients had incomplete treatment: 3.4% (5/149, ASAQ) and 4.7% (7/150 AL) respectively. This was either due to premature study-discontinuation (1/149 ASAQ incorrect enrolment; 1/150 AL repeated vomiting of dose, SAE), missed visits for dose-intake (2/150 AL, n = 1 one evening dose missed, n = 2 two evening doses missed), or spitting-out/wasting (part) of a dose (4/149 ASAQ arm; 4/150 AL arm). Ninety-two patients (61.7%, ASAQ) and 104 patients (69.3%, AL) completed 42 days of follow-up. Premature study discontinuations were mainly due to recurrent parasitaemia before day 42 (Figure 1). No patient was lost to follow-up, two caregivers had withdrawn consent for two participating children (day 3 and day 21, respectively, AL). Three patients were prematurely discontinued due to a serious adverse event (SAE): one severe pneumonia (day 3, ASAQ), one severe malaria (*P. falciparum* re-infection, day 30, ASAQ), and one severe vomiting (onset severe vomiting on day 1, withdrawn and hospitalized after repeated vomiting of day 1 evening study dose, AL). All SAEs had outcome fully recovered. Major protocol deviations and pre-mature study discontinuations other than AE or recurrence of parasitaemia lead to exclusion from the per-protocol population for seven (4.7%, ASAQ) and 24 (16%, AL) patients, respectively (Figure 1).

**Table 1:** Demographic and parasitological baseline characteristics by treatment arm, mITT population

	**ASAQ**	**AL**
	**N = 149**	**N = 150**
Sex (male), N (%)	86 (57.7)	89 (59.3)
Age (months), mean (sd)	37.2 (13.7)	37 (13.6)
Weight (kg), mean (sd)	12.8 (2.5)	12.9 (2.6)
Axillary temperature (°C), mean (sd)	37.1 (1.1)	36.9 (0.8)
Haemoglobin (g/dl) ^*^, mean(sd)	9.2 (1.6)	9.3 (1.5)
*P. falciparum* trophozoites/μl, geometric mean, (95% CI)	20020	19152
	(15878–25244)	(15386–23839)
*P. falciparum* trophozoite density/μl, N (%)		
< 2,000	0	0
2,000 - < 50,000	105 (70.5)	113 (75.3)
50,000 - < 100,000	21 (14.1)	22 (14.7)
100,000 - < 150,000	15 (10.1)	8 (5.3)
150,000 - < 200,000	6 (4.0)	7 (4.7)
> 200,000	2 (1.3)	0
*P. falciparum* gametocytes carriage, N (%)	22 (14.8)	22 (14.7)

*Sd* standard deviation, *CI* confidence interval. * Hb measures by Act5diff, Beckman Coulter®.

### Primary efficacy outcomes

**Figure 2:**
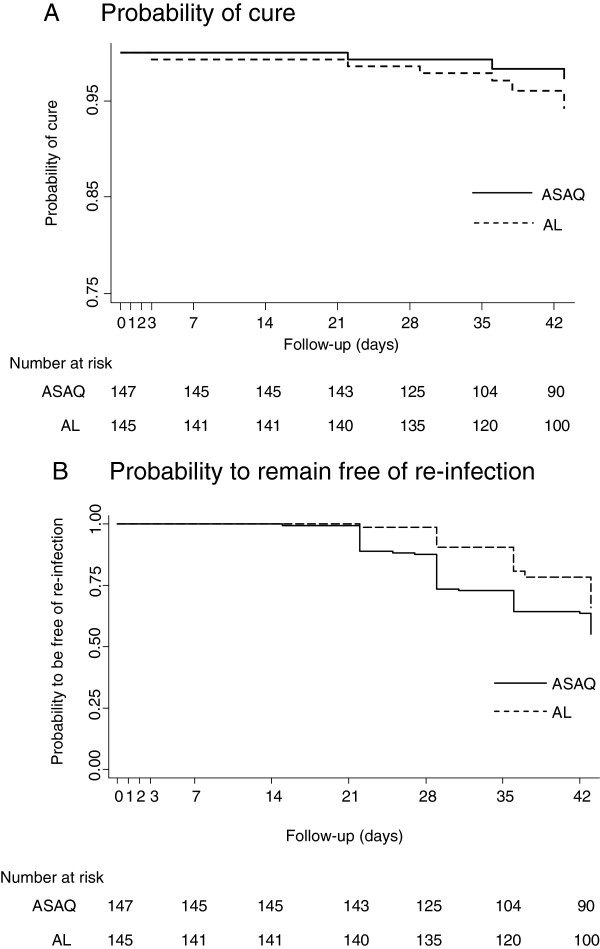
**Kaplan-Meier survival curves.** Modified intention to treat (mITT) population. Full line (ASAQ arm), dashed line (AL arm). **(A)** genotyping-adjusted probability to be cured. **(B)** probability to remain free of re-infection.

Treatment outcomes and endpoint classifications for efficacy analyses are displayed in Table 2 (mITT/KM analysis) and Table 3 (PP/% cured analysis). One patient in the ASAQ arm and three in the AL arm experienced treatment failure up to day 28, amounting to a total of three (ASAQ) and six (AL) failures by day 42 (Table 2) (mITT). All treatment failures were LPFs (days 21–42) with exception of one ETF (day 2, AL). All failures had completed the study treatment according to schedule, except one LPF (skipped both day 1 doses, completed regimen on day 3, AL arm). Among patients with recurrent parasitaemia, seven had missing or inconclusive genotyping results (n = 2 ASAQ; n = 5 AL) (excluded from genotyping-adjusted analyses). The day 42 genotyping-adjusted cumulative cure rate estimate (mITT/ KM) was 97.3% [CI 95%: 91.6-99.1] (ASAQ) and 94.2% [88.1-97.2] (AL), respectively (Figure 2A, Table 4). The cure rate difference (AL-ASAQ) was −3.1% with an upper limit 95% CI of 1.2%, demonstrating non-inferiority of ASAQ to AL. Results were similar for percentage cured on the PP population (PP/%) (Table 4).

**Table 2:** Efficacy endpoint classification at day 28 and day 42, - mITT/KM analysis

**Treatment outcome**	**Day 28**		**Day 42**		
mITT population, N (%)	ASAQ	AL	ASAQ	AL	KM analysis
	N = 149	N = 150	N = 149	N = 150	
ACPR	104 (69.8)	123 (82.0)	77 (51.7)	82 (54.7)	Success
ETF	0	1 (0.7)	0	1 (0.7)	Failure
LCF or LPF					
- *P. falciparum* recrudescence	1 (0.7)	2 (1.3)	3 (2.0)	6 (4.0)	Failure
- *P. falciparum* re-infection	39 (26.2)	13 (8.7)	64 (43.0)	45 (30.0)	Censored*
- undetermined or missing PCR	2 (1.3)	2 (1.3)	2 (1.3)	5 (3.3)	Excluded*
Non-*P. falciparum* malaria infection **	0	3 (2.0)	0	5 (3.3)	Censored
No treatment outcome					
- discontinued for SAE ^#^	1 (0.7)	1 (0.7)	1 (0.7)	1 (0.7)	Censored
- withdrawal after incorrect enrolment ^##^	1 (0.7)		1 (0.7)		Censored
- unable to complete follow-up visits		1 (0.7)		1 (0.7)	Censored
- withdrawal of consent		2 (1.3)		2 (1.3)	Censored
- ≥ 2 consecutive visits not performed	1 (0.7)	2 (1.3)	1 (0.7)	2 (1.3)	Censored
Patients analysed					
Genotyping-adjusted analysis	N = 147	N = 148	N = 147	N = 145	
Genotyping-unadjusted analysis	N = 149	N = 150	N = 149	N = 150	

*mITT* modified Intention To Treat, *KM* Kaplan-Meier, *PP* per protocol, *ACPR* Adequate Clinical and Parasitological Response, *ETF* Early Treatment Failure, *LPF* Late Parasitological Failure, *LCF* Late Clinical Failure, *PCR* Polymerase Chain Reaction, *SAE* Serious Adverse Event.

* coded as Failure in genotyping -un-adjusted analysis.

** *Plasmodium malariae* infections.

^#^ n=1 patient with SAE (= severe malaria due to re-infection with *Pf* malaria, ASAQ, day 30) is not listed here among SAE, but instead listed among the re-infections (= efficacy endpoint).

^##^ n=1 withdrawn following incorrect enrolment on day 0 (=asexual parasites density outside the eligibility range).

**Table 3:** Efficacy endpoint classification at day 28 and day 42, - PP / percent cured analysis

**Treatment outcome**	**Day 28**		**Day 42**		
PP population, N (%)	ASAQ	AL	ASAQ	AL	% cured analysis
	N = 142	N = 126	N = 142	N = 126	
ACPR	99 (69.7)	108 (85.7)	75 (52.8)	71 (56.3)	Success
ETF	0 (0)	1 (0.8)	0 (0)	1 (0.8)	Failure
LCF or LPF					
- *P. falciparum* recrudescence	1 (0.7)	2 (1.6)	3 (2.1)	5 (4.0)	Failure
- *P. falciparum* re-infection	39 (27.5)	11 (8.7)	61 (43.0)	40 (31.7)	Excluded*
- undetermined or missing PCR	2 (1.4)	2 (1.6)	2 (1.4)	5 (4.0)	Excluded*
Non-P. falciparum malaria infection **	0 (0)	2 (1.6)	0 (0)	4 (3.2)	Excluded
No treatment outcome					
- discontinued for SAE	1 (0.7)	0 (0)	1 (0.7)	0 (0)	Excluded
Patients analysed					
Genotyping-adjusted analysis	N = 100	N = 111	N = 78	N = 77	
Genotyping-unadjusted analysis	N = 141	N = 124	N = 141	N = 122	

*mITT* modified Intention To Treat, *KM* Kaplan-Meier, *PP* per protocol, *ACPR* Adequate Clinical and Parasitological Response, *ETF* Early Treatment Failure, *LPF* Late Parasitological Failure, *LCF* Late Clinical Failure, *PCR* Polymerase Chain Reaction, *SAE* Serious Adverse Event. * coded as Failure in genotyping-unadjusted analysis. ** *Plasmodium malariae* infections.

**Table 4:** Genotype adjusted and non-adjusted day 28 and day 42 cure rates, and day 42 non-inferiority analyses

	**KM cure rate estimates**		**Observed proportion cured**	
	**mITT population**		**PP population**	
**Day 28**	ASAQ	AL	ASAQ	AL
**Genotyping-adjusted**				
% cured [95% CI]	99.3 [95.1–99.9]	97.9 [93.6–99.3]	99.0 [94.6–100]	97.3 [92.3–99.4]
**Genotyping-unadjusted**				
% cured [95% CI]	71.2 [63.1– 77.9]	87.5% [80.9–91.9]	70.2 [61.9–77.6]	87.1 [79.9–92.4]
**Day 42**				
**Genotyping-adjusted**				
% cured [95% CI]	97.3 [91.6–99.1]	94.2 [88.1–97.2]	96.2 [89.2–99.2]	92.2 [83.8–97.1]
% difference ^#^ (UL 95% CI*)	**−3.1 (1.2)**		**−3.9 (2.2)**	
**Genotyping-unadjusted**				
% cured [95% CI]	52.7 [44.3–60.5]	59.5 [50.9–67.1]	53.2 [44.6–61.6]	58. 2 [48.9–67.1]

^#^ Percent difference in day 42 genotyping-adjusted cure rate: AL arm day 42% cured- ASAQ arm day 42% cured. * According to the method of Blackwelder: one-sided 95% upper limit (UL) CI of a two-sided 90% CI around the difference of efficacy of cure rates. The CI was assessed by Wald method.

### Secondary efficacy outcomes

**Figure 3:**
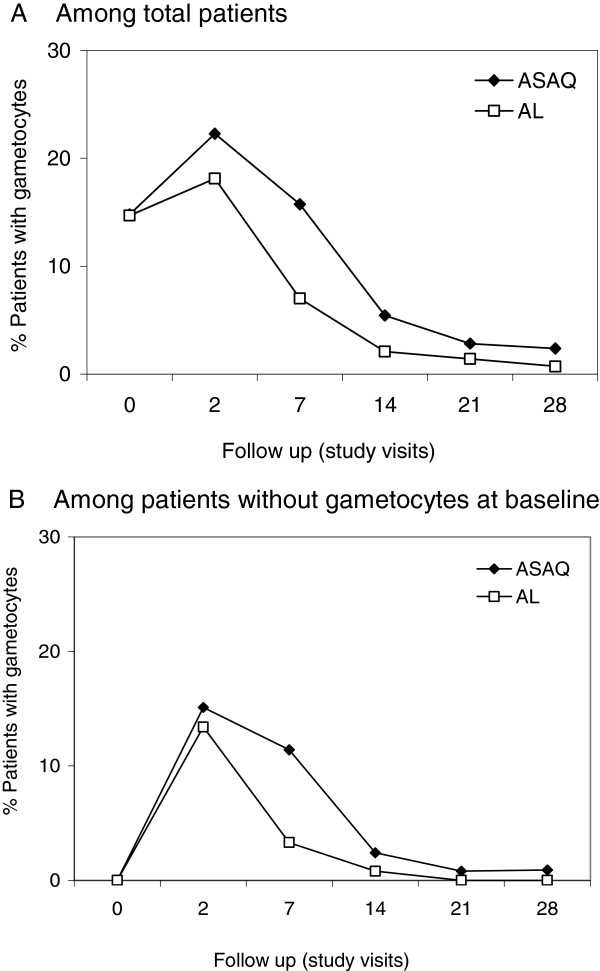
***Pf *Gametocyte carriage by malaria blood smear up to day 28, by treatment arm.***Pf* = *Plasmodium falciparum.***(A)** Percentage of patients with *Pf* gametocytes by blood smear among total patients by follow up day, by treatment arm, mITT population. **(B)** Percentage of patients with *Pf* gametocytes by blood smear among patients who had no gametocytes on day 0, by follow up day, by treatment arm, mITT population.

Day 28 genotyping-adjusted cure rate estimates were 99.3% [95.1-99.9] (ASAQ) and 97.9% [93.6-99.3] (AL) (mITT/KM) (Table 4). Genotyping-unadjusted cure rates were 71.2% [63.2-77.9] (ASAQ) and 87.5% [80.9-91.9] (AL) at day 28, and 52.7% [44.3-60.5] (ASAQ) and 59.5% [50.9-67.1] (AL) at day 42 (mITT/ KM), respectively. Comparable results were obtained in PP/% analysis (Table 4). The Kaplan Meier estimated probability to remain free of re-infection was 0.55 [95% CI 0.46-0.63] in the ASAQ arm and 0.66 [0.57-0.73] in the AL arm (log-rank test, p = 0.017) (Figure 2B). The median time to re-infection was 29 days (mITT, ASAQ arm, IQR: 24–36) and 36 days (mITT, AL arm, IQR: 29–43) (p < 0.001). Results on PP population were similar. Day 2 parasite clearance was 98% in both treatment arms (145/148 ASAQ; 146/149, AL), and reached 100% (147/147, ASAQ) and 99.3% (148/149, AL) by day 3 (mITT population). One patient remained parasitaemic on day 3 (ETF, AL). Gametocyte carriage peaked on day 2 with 22.3% (33/148, ASAQ) and 18.12% (27/149) and decreased to 2.4% (3/127, ASAQ) and 0.7% (1/139, AL) by day 28 (mITT) (Figure 3). Among patients who had no gametocytes at baseline, gametocyte carriage also peaked on day 2 and declined to 0.9% (1/108, ASAQ) and 0% (0/117, AL) by day 28 (Figure 3). The day 28 genotyping-adjusted cure rates among patients >5 years (parallel tolerability trial [15]) were 98.4% [96.7-99.2] (ASAQ) and 100% (AL) (mITT/KM), and 98.3% [97.0 – 99.5] (ASAQ) and 100% (AL) (PP/% cured), respectively. Few patients had presented with fever (> 37.5°C) at enrolment (n = 49 ASAQ arm, n = 29 AL arm, mITT population). For the majority of children history of fever in the last 48 hours before enrolment was reported. Among patients with fever on day 0 in the ASAQ arm, 98% were fever-free on day 1 and 2, and 100% fever-free on day 3. Fever clearance in the AL arm was 100% by day 1.

### Day 7 blood concentrations of AQ, DEAQ, LF

Blood concentrations of amodiaquine (AQ), desethylamodiaquine (DEAQ) (ASAQ arm) and lumefantrine (LF) (AL arm) were assessed from day 0 (baseline) and day 7 blood samples. Amodiaquine-intake shortly before study inclusion (protocol violation) was evident for six patients (ASAQ arm) who had quantifiable day 0 AQ levels (range: 3.6 – 34 ng/ml). Three of these had also high DEAQ levels (>200 ng/ml). Seventy-eight patients had low DEAQ blood concentrations on day 0 (range: 5.0 -142 ng/ml), indicating a previous treatment with AQ (no violation). On day 7, three patients had DEAQ values below the LOQ (<2.5 ng/ml), suggestive of sub-optimal drug intake or mal-absorption. All three patients had completed the drug intake according to schedule and had day 42 efficacy outcome ACPR. Five patients also had detectable AQ on day 7, indicative of AQ intake before sample collection (protocol violation) (range: 2.6-4.2 ng/ml). No LF blood concentrations were detected in the AL arm at baseline. On day 7, 33 patients had non-quantifiable LF concentrations (< LOQ, 200 ng/ml). These were set as 100 ng/ml (=LOQ/2) by convention for all blood concentration analyses, since the LOQ was considered very high.

The median day 7 DEAQ blood concentration of all patients in the ASAQ arm was 423 ng/ml (IQR 314, 602; 137/149 samples available and quantifiable, mITT). The median day 7 LF concentration was 310 ng/ml (IQR 200, 447;139/150 samples available and quantifiable, mITT, AL arm). The recrudescences in the ASAQ arm had day 7 DEAQ blood concentrations of 242, 401 and 688 ng/ml, respectively. Recrudescences in the AL arm had day 7 LF concentrations of 271, 447 and 515 ng/ml, respectively (n = 1 no sample available, n = 1 LF < LOQ). Comparison of day 7 blood concentrations between patients with day 42 efficacy outcome ACPR versus recrudescence revealed no significant differences for DEAQ (ASAQ arm, p = 0.641) or LF (AL arm, p = 0.775), respectively (Table 5). Similarly, comparison of day 7 DEAQ drug concentrations between ACPR versus re-infections or ACPR versus all *P. falciparum* recurrences combined were not significantly different (ASAQ arm: p = 0.209, p = 0.205, Table 5). Day 7 LF blood concentrations were significantly lower in patients with re-infection (p = 0.022) or in patients with any type of *P. falciparum* recurrences (p = 0.015) when compared to ACPRs (AL arm, Table 5). Similar results were obtained when blood concentrations of patients with indication of recent AQ intake on day 0 or day 7 (protocol violation) or low DEAQ on day 7 (ASAQ arm), and patients with day 7 LF values < LOQ (AL arm) were excluded from all day 7 blood concentration comparison analyses.

**Table 5:** Day 7 desethylamodiaquine- and lumefantrine blood concentrations by day 42 efficacy outcome and treatment arm (mITT)

**Study arm**	**Compound**	**Patients n/N **^**#**^	**Day 42 efficacy endpoint**	**Blood concentrations [ng/ml] median (IQR)**	**Wilcoxon rank-sum (Mann–Whitney) test ϵ**
**ASAQ**	**DEAQ**	73/77	ACPR	456 (167, 1727)	
		3/3	recrudescence^1^	402 (242, 688)	p = 0.641
		59/64	*P. falciparum* re-infection	410 (308, 525)	p = 0.209
		2/2	Recurrence with un-determined or missing PCR	525 (277, 772)	
		64/69	Recurrences combined^2^	407 (303, 525)	p = 0.205
**AL**	LF *	80/82	ACPR	356 (221, 547)	
		4/6	recrudescence^1^	359 (186, 481)	p = 0.775
		43/45	*P. falciparum* re-infection	295 (200, 363)	p = 0.022
		5/5	Recurrence with un-determined or missing PCR	254 (100, 262)	
		52/56	Recurrences combined^2^	276 (150, 373)	p = 0.015

^#^n = Number of patients with blood concentration sample available and quantifiable among total N.

*n = 33 day 7 LF values were < LOQ. Values were set as LOQ/2 (=100 ng/ml).

^1^Recrudescence = LPF + LCF.

^2^Recurrences combined = Recrudescence, re-infection and recurrence with undetermined or missing PCR combined.

ϵComparison between day 7 drug concentrations of patients with day 42 efficacy outcome ACPR versus recrudescence, ACPR versus re-infection, ACPR versus *Pf* recurrences combined.

*AQ* amodiaquine, *LF* lumefantrine, *DEAQ* desethylamodiaquine, *mITT* modified Intention To Treat population, *ACPR* Adequate Clinical and Parasitological Response, *Pf* Plasmodium falciparum, *PCR* Polymerase Chain reaction.

### Day 0 blood concentrations of other anti-malarials

Stored day 0 serum samples were retrospectively analysed for detectable concentrations of other anti-malarials (Table 6, mITT population). At baseline 17% (ASAQ arm) and 10% (AL arm) of children had detectable concentrations of chloroquine, 8.1% (ASAQ) and 4.0% (AL) had detectable concentrations of sulphadoxine, 2% (ASAQ) and 4.7% (AL) had detectable quinine, and 1.3% (ASAQ) and 0.7% (AL) had detectable pyrimethamine, respectively. Median drug concentrations are tabulated (Table 6).

**Table 6:** Day 0 serum concentration of other anti-malarial drugs, mITT population

**Study arm**	**ASAQ**		**AL**	
	N = 149*		**N = 150**	
**Antimalarial drug detected**	**Patients**	**Serum concentration [ng/ml] median (range)**	**Patients**	**Serum concentration [ng/ml] median (range)**
	**n (%)**		**n (%)**	
Pyrimethamine	2 (1.3)	73 (10–135)	1 (0.7)	408 (408)
Sulphadoxine	12 (8.1)	49 (12–74323)	6 (4.0)	148 (22–123598)
Chloroquine	25 (16.9)	127 (10–559)	15 (10.0)	82 (11–572)
Quinine	3 (2.0)	32 (13–39)	7 (4.7)	413 (13–8511)

*samples available for 148 of 149 patients.

## Discussion

Since 2003, AS + AQ loose dose has been adopted as first-line treatment for uncomplicated falciparum malaria in Liberia. Published information on the efficacy of AS + AQ or other forms of ACT in the country are currently limited to only one observational study on the efficacy of artemether-lumefantrine in Tubmanburg and Harper region [14]. We report here on the *in vivo* efficacy of two ACTs, ASAQ fixed-dose-combination (FDC) and artemether-lumefantrine (AL) among 6–59 month old children with uncomplicated falciparum malaria in Nimba County, a highly Pf malaria endemic area in Northern Liberia. The day 42 genotyping-adjusted cure rate estimates of ASAQ and AL reached 97.3% and 94.2% respectively (mITT / KM). Both treatments were well above the WHO-recommended 90% threshold for treatments in use [10]. ASAQ FDC was shown to be non-inferior to AL after 42 days of follow up in two parallel analytic approaches. Few treatment failures were detected, but > 50% occurred after day 28 in both study arms, arguing for 42 days follow up when monitoring these two treatments. In the parallel tolerability trial conducted among patients > 5 years, an age group likely partially immune, day 28 genotyping-adjusted cure rates of ASAQ and AL were also high (> 95%). Though the limited malaria blood smear follow up in this second trial should be considered. Both ASAQ and AL were also well tolerated among children six to 59 month and among the older age group (>5 years), as described elsewhere in more detail [15].

Overall, efficacy results obtained in the present studies were very good for both ACTs. This was also encouraging for the utility of ASAQ FDC in a geographical setting where amodiaquine monotherapy and AS + AQ (non fixed formulation, i.e. blister packs) were used for several years. Notably however, anecdotal reports suggested that the prescription of AS + AQ loose dose provided the opportunity to disregard the amodiaquine compound, referring to patients’ and providers’ concern on tolerability related to prescriptions of higher dose amodiaquine monotherapy in the past [21]. The present findings were also in line with efficacy results recently reported for ASAQ FDC and AL in Western-, Central- and Southern African regions or India [6-9,22-24]. A recent study also demonstrated good effectiveness of both ASAQ FDC and AL among children ≤ 5 years following unobserved treatment in Benin [25]. Taken together these findings also open the possibility of using several efficacious and well tolerable forms of ACT in the same country, which may decrease drug pressure. As anti-malarial drug efficacy may vary within regions and even within countries, ideally therapeutic efficacy studies would be performed regularly at representative sentinel sites if this is feasible.

The proportion of parasitaemic patients on day 3 has been reported as an interesting indicator for monitoring artemisinin resistance [26]. Although this indicator requires large sample sizes and accurate timing of sample collection for precise estimates and is dependent on pre-treatment parasite density, the low proportion of patients parasitaemic at day 3 for both ASAQ and AL in the present study was nevertheless reassuring. The use of more accurate estimations of parasite clearance rates with recently developed tools should be preferred but demand repeated parasitaemia measures [27]. Since sub-optimal drug exposure is considered among the main reasons for treatment failure, the day 7 blood concentration of the slowly eliminated artemisinin-partner drugs was proposed as a simplified and valid pharmacokinetic predictor of treatment outcome [28]. Day 7 DEAQ or LF bloods concentrations in the present study were overall close to those reported in two previous studies using whole blood samples on filter paper [19,29]. No evidence was found that treatment failure in either study arm was linked with inadequate drug exposure when comparing day 7 DEAQ or LF drug levels, respectively, among patients with ACPR versus recrudescence. The comparison for recrudescences was however limited by the few treatment failures. Notably day 7 LF concentrations were significantly lower among patients with re-infection than among patients with ACPR, while this was not the case for day 7 DEAQ concentrations. No direct correlation between day 7 drug levels and re-infection has been established yet, and additional measurements beyond day 7 would be needed for a more robust analysis of a potential correlation. Fever clearance was good in both treatment arms among the few children who had presented with temperatures >37.5°C on day 0. Gametocyte carriage was reduced significantly during follow up. It seemed slightly slower in the ASAQ arm than in the AL arm, as indicated mainly by a higher percentage of gametocyte carriage on day 7.

The overall *P. falciparum* re-infection rate was high in the present study, emphasizing the burden of malaria among the non-immune < 5 year olds in Nimba County. Re-infection rates were higher in the ASAQ arm than the AL arm, and AL conferred a longer secondary prophylactic effect than ASAQ with a significantly lower median time to re-infection in the ASAQ arm. The current WHO treatment protocol emphasizes the curative effect of the anti-malarial treatment, i.e. a fast elimination of the parasite [2]. A potentially increased risk of resistance development has also been discussed for artemisinin partner compounds that confer longer post-treatment prophylaxis [30,31], and the benefit of the prophylactic effect of an anti-malarial may also need to be compared against other essential elements, such as fast parasite elimination, good tolerability, easy administration and good compliance.

In 2001 high resistance to chloroquine and sulphadoxine-pyrymethamine (SP), the respective first- and second-line treatments in the country at the time, were reported in the Harper region, Liberia [32]. A second study showed that amodiaquine (AQ) monotherapy still had relatively good efficacy [32,33]. In 2003, AS-AQ combination therapy (provided as co-blister-packed separate tablets) was adopted as first-line treatment for uncomplicated falciparum malaria in Liberia. Non-ACT, such as chloroquine and amodiaquine are however still available and used by the population in Liberia [21,34]. In the present study, assessment of day 0 serum concentrations of various anti-malarials indicated that a considerable proportion of children had recent anti-malarial intake, mainly chloroquine and sulphadoxine. These findings are not unexpected in a highly endemic setting, and are unlikely to have impacted much on the efficacy results. However, ongoing use of non-ACT, including chloroquine which can evoke cross-resistance to amodiaquine, re-emphasizes the need to ensure wide–spread and continuous access to the first-line ACT while removing access and use of non-ACT. Following the introduction of ASAQ FDC as first-line treatment in 2010, the most recent Liberian Malaria Indicator survey conducted in 2011 reported that for about seven out of 10 children who received an anti-malarial treatment, caretakers indicated the use of an ACT (referring to “the new malaria medicine”, i.e. ASAQ FDC) [21]. These findings are encouraging and should be supported with ongoing efficacy monitoring.

## Conclusion

ASAQ and AL were both highly efficacious treatments for uncomplicated falciparum malaria in Nimba County. These findings support both drugs as treatment options. Since the end of 2010, ASAQ FDC was adopted as national first-line treatment in Liberia. As for all malaria endemic settings, anti-malarial treatment monitoring should continue on a regular basis, ideally including recently developed early indicators of emerging artemisinin resistance.

## Competing interest

The authors declared no competing interests.

## Authors’ contributions

BS overall trial coordination, participation in study design and protocol, data analysis plan, study documents, drafting of the manuscript. PV field trial coordination, medical coordination, trial team supervision, supervision of data collection. EB support study design, coordination of data management, data analysis plan, data analysis, revision of the manuscript. CM field laboratory coordination, laboratory standard operating procedures and data collection. RS field coordination during study preparation, writing of administrative and clinical standard operating procedures, preparation of study site, staff training, trial implementation. LP data management, support data analysis plan, data analysis, revision of the manuscript. MD and JB coordination and interpretation of the malaria parasite genotyping. TS support field trial coordination, team supervision and data collection. YMZ and JJJ technical support to all study steps, participation in study implementation and training. EC support study initiation and study design, technical support during study conduct. PH anti-malarials baseline serum analysis. VJ development and conduct of blood concentration analyses of artemisinin-partner compounds. GC technical advise to protocol development and study preparation, support field training and study conduct, review of study documents, coordination of study monitors and data monitoring committee, revision of the manuscript. JR. study initiation, technical and scientific advise to study protocol-, study preparation and conduct, review of study documents, revision of the manuscript. EAA study initiation, study design and protocol, scientific and medical advise to all steps of the study, revision of study documents, support data analysis plan, revision of the manuscript. PJG study initiation, study design and protocol, scientific and medical advise to all steps of the study, review of study documents and medical review of adverse events, support data analysis plan, revision of the manuscript. All authors read and approved the manuscript.

## Supplementary Material

Additional file 1 Distribution of patients’ age and weight by weight-based drug-dosing group and by study arm (mITT population). Click here for file
