# Supplementary material for: Efficacy of artesunate-amodiaquine and artemether-lumefantrine fixed-dose combinations for the treatment of uncomplicated Plasmodium falciparum malaria among children aged six to 59 months in Nimba County, Liberia: an open-label randomized non-inferiority trial
Source: Malar J. 2013 Jul 17;12:251. doi: 10.1186/1475-2875-12-251 (PMC3728070; doi:10.1186/1475-2875-12-251)
Supplement: Additional file 1 — Distribution of patients’ age and weight by weight-based drug-dosing group and by study arm (mITT population). [file 1475-2875-12-251-S1.doc]

Additional File 1: Distribution of patients’ age and weight by weight-based drug-dosing group and by study arm (mITT population).

| Study arm | Weight group for drug dosing | Patients  n (%) | Weight [kg]  median (range) | Age [months]  median (range) |
| --- | --- | --- | --- | --- |
| ASAQ  N=149 | 5-<9kg | 8 (5.4) | 8.3 (7.8, 8.7) | 14.8 (8.5, 17.2) |
|  | 9-<18kg | 137 (91.9) | 12.8 (9, 17.8) | 37.7 (11.8, 69.2) |
|  | 18-35kg | 4 (2.7) | 18.6 (18, 20) | 50.8 (41.3, 59.3) |
|  | | | | |
| AL  N=150 | 5-<15kg | 118 (78.7) | 12 (7, 14.9) | 33.9 (8.9, 59.1) |
|  | 15-<25kg | 32 (21.2) | 16.1 (15, 21) | 50.3 (30, 60.1) |
|  | 25-34kg | - | - |  |
